# Supplementary material for: Prioritized SNP Selection from Whole-Genome Sequencing Improves Genomic Prediction Accuracy in Sturgeons Using Linear and Machine Learning Models
Source: Int J Mol Sci. 2025 Jul 21;26(14):7007. doi: 10.3390/ijms26147007 (PMC12295944; doi:10.3390/ijms26147007)
Supplement: Supplementary file 1 [file ijms-26-07007-s001.zip › ijms-3735047-supplementary.pdf]

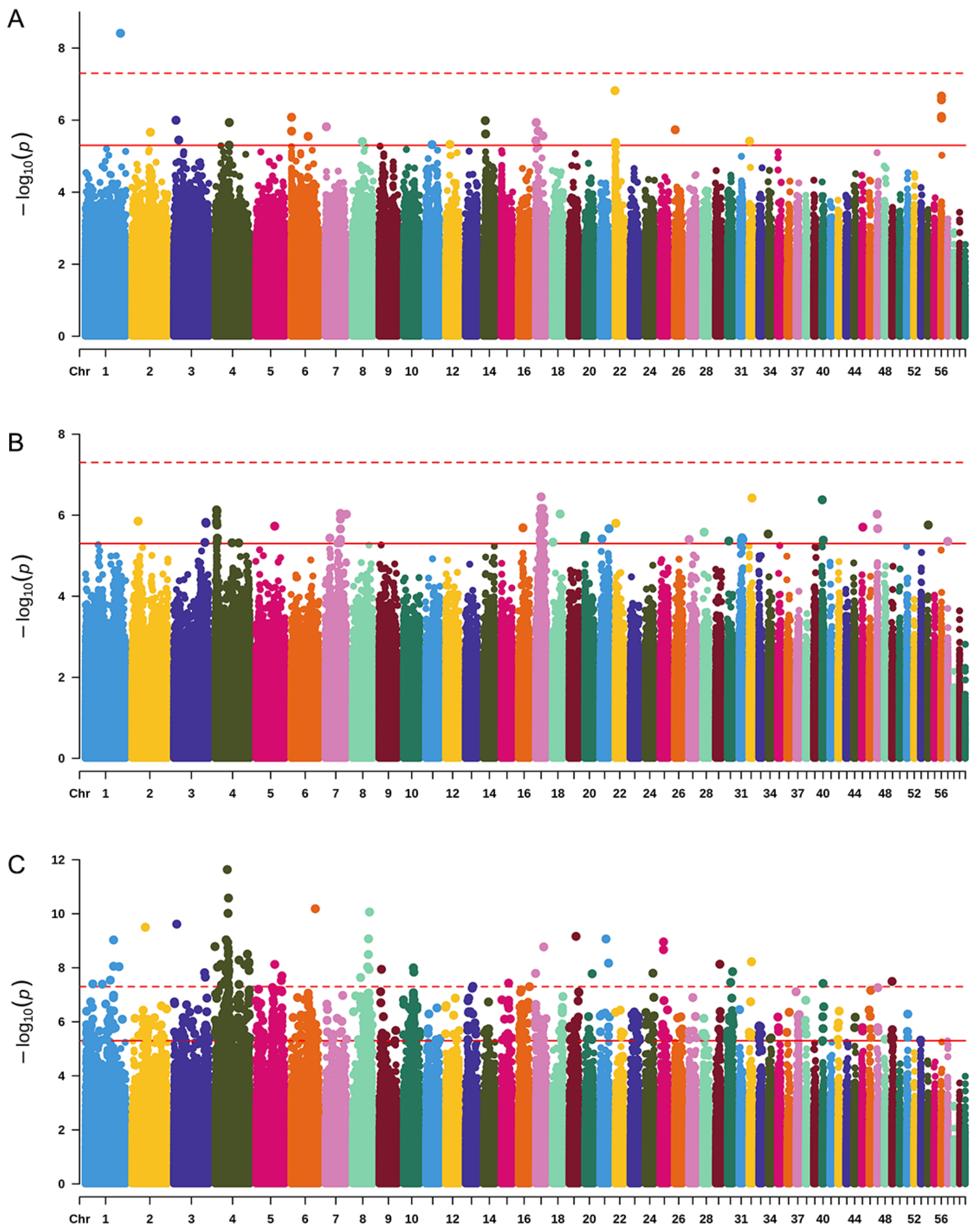

Figure S1 Manhattan plots of genome-wide association studies for (A) caviar yield, (B) caviar color, and (C) body weight in the Russian sturgeon population. In the Manhattan diagram, the dashed and solid lines indicate the genome-wide (5×10^{-8}) and suggestive (5×10^{-6}) significance threshold, respectively.

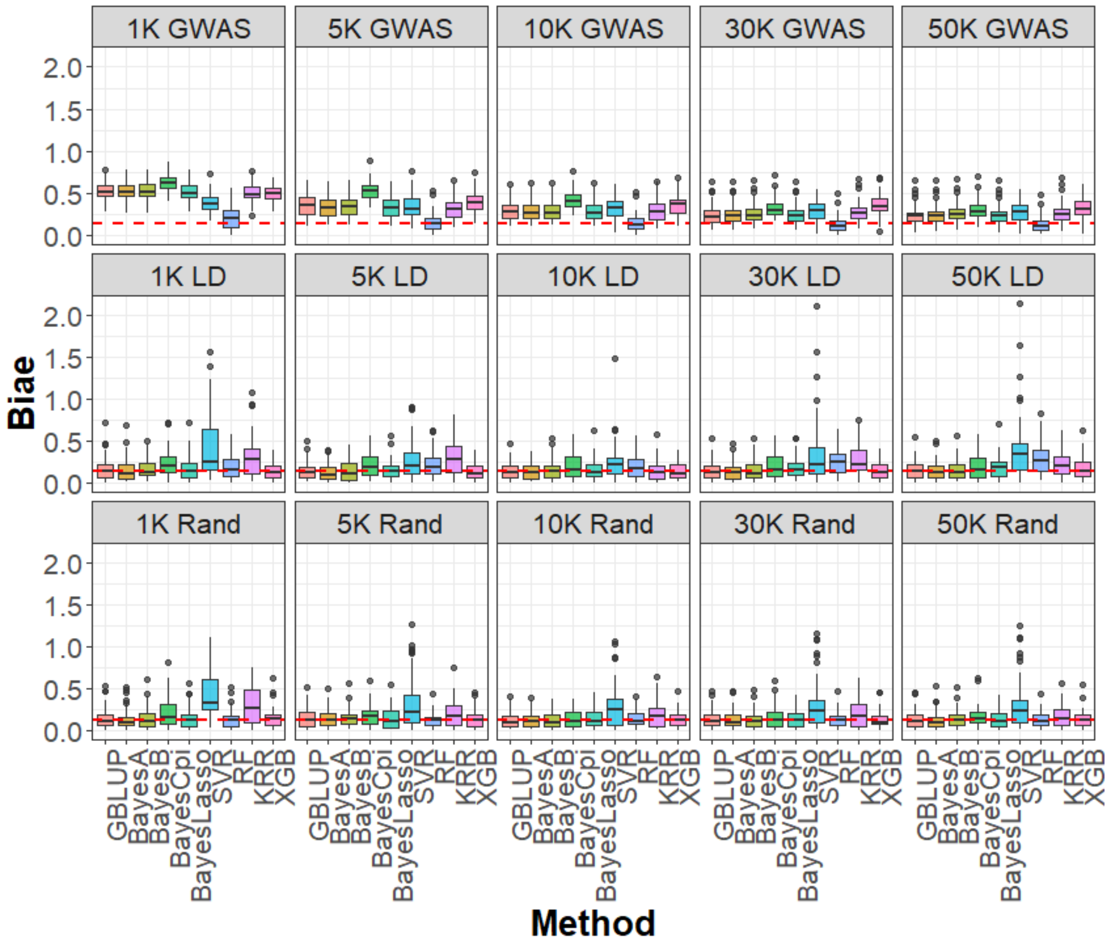

Figure S2 Bias of genomic prediction for caviar yield based on GWAS, linkage disequilibrium (LD), and random SNP selection strategies across different SNP densities, using linear and machine learning models.

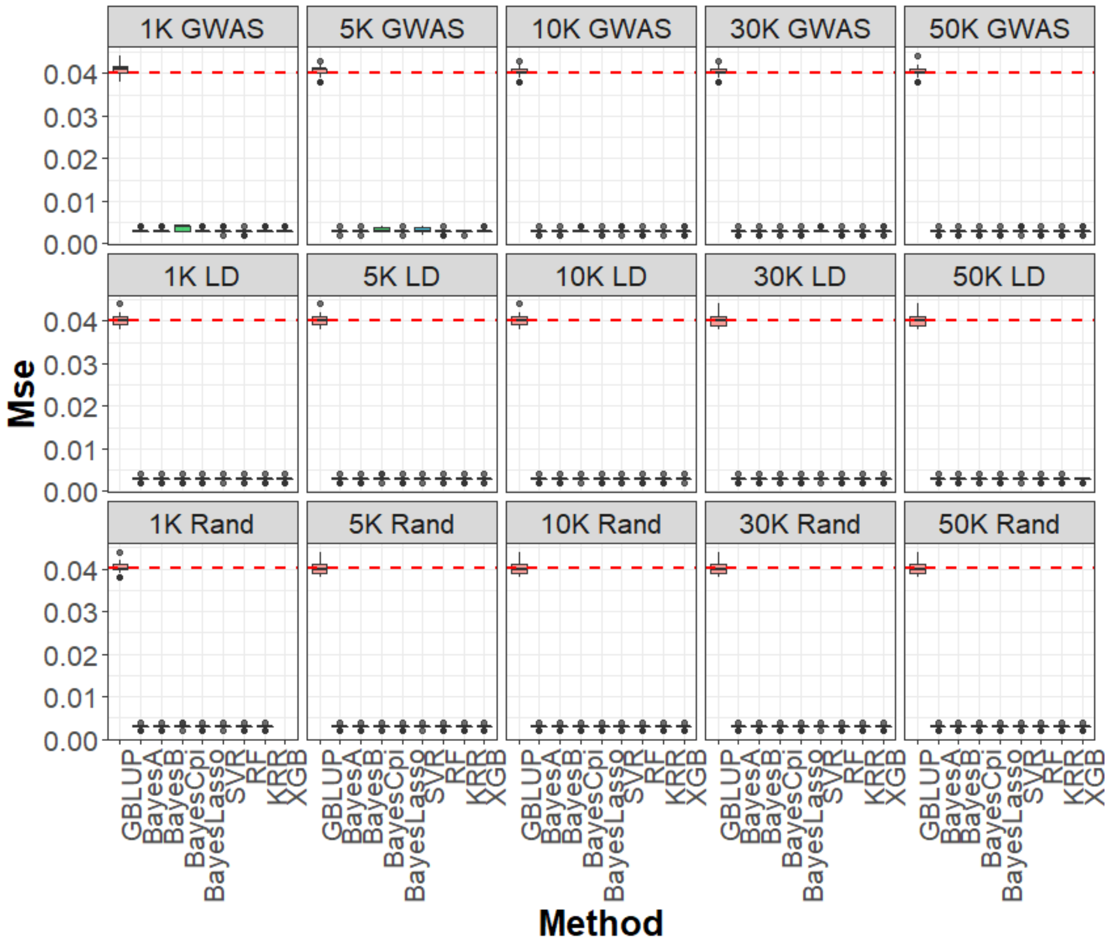

Figure S3 Mean squared error (Mse) of genomic prediction for caviar yield based on GWAS, linkage disequilibrium (LD), and random SNP selection strategies across different SNP densities, using linear and machine learning models.

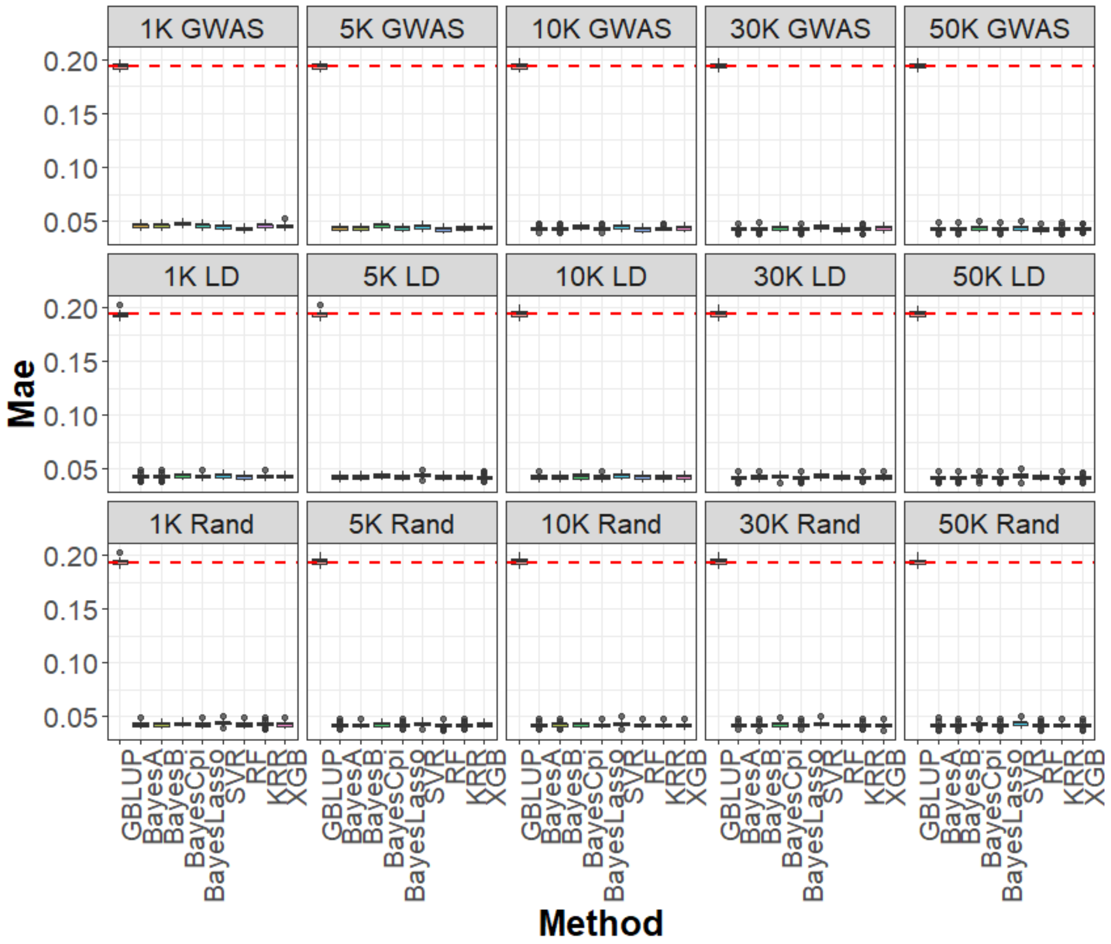

Figure S4 Mean absolute error (Mae) of genomic prediction for caviar yield based on GWAS, linkage disequilibrium (LD), and random SNP selection strategies across different SNP densities, using linear and machine learning models.

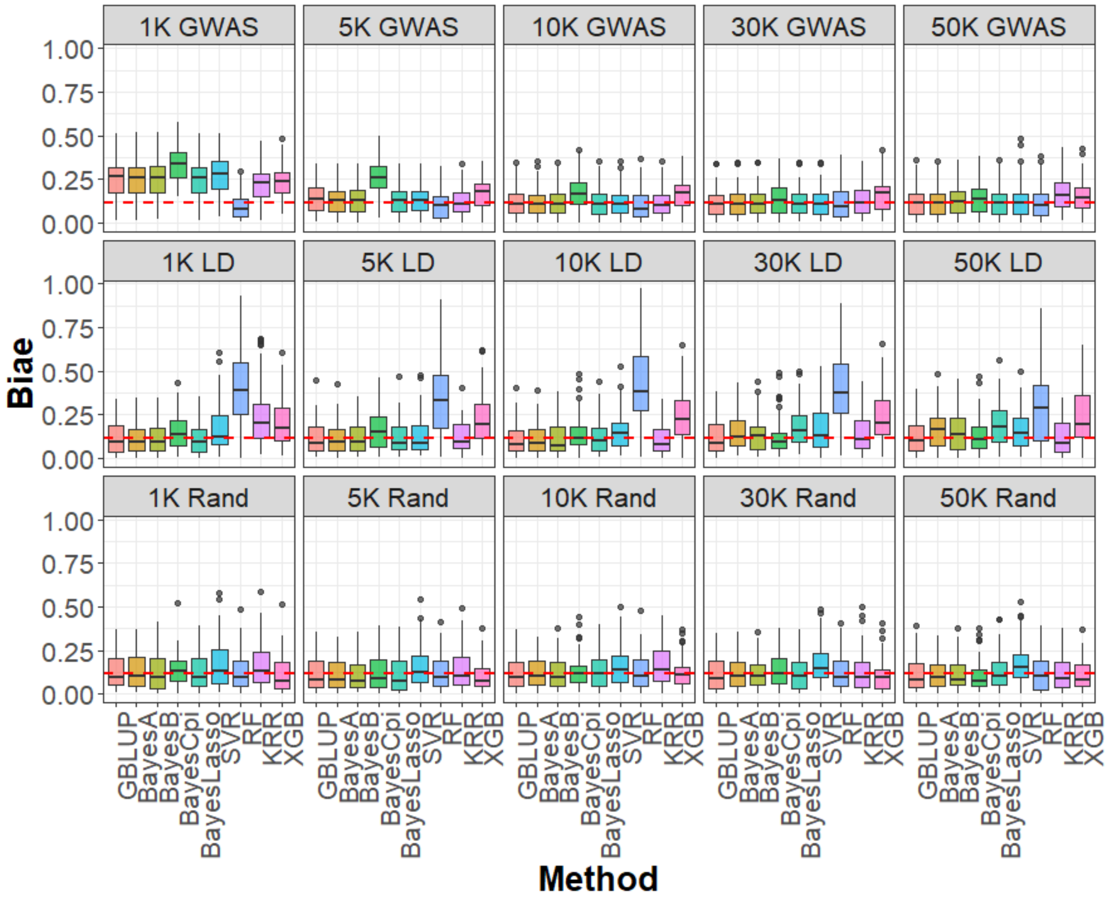

Figure S5 Bias of genomic prediction for caviar color based on GWAS, linkage disequilibrium (LD), and random SNP selection strategies across different SNP densities, using linear and machine learning models.

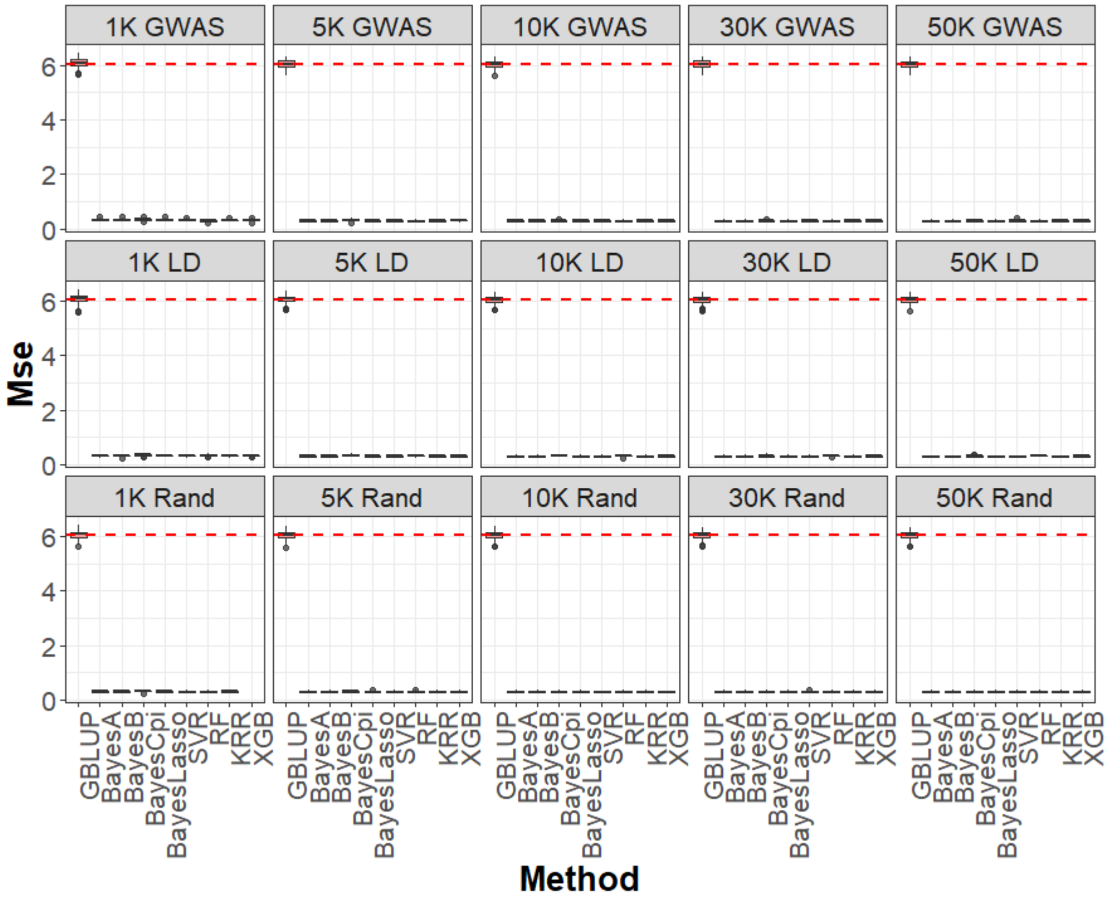

Figure S6 Mean squared error (Mse) of genomic prediction for caviar color based on GWAS, linkage disequilibrium (LD), and random SNP selection strategies across different SNP densities, using linear and machine learning models.

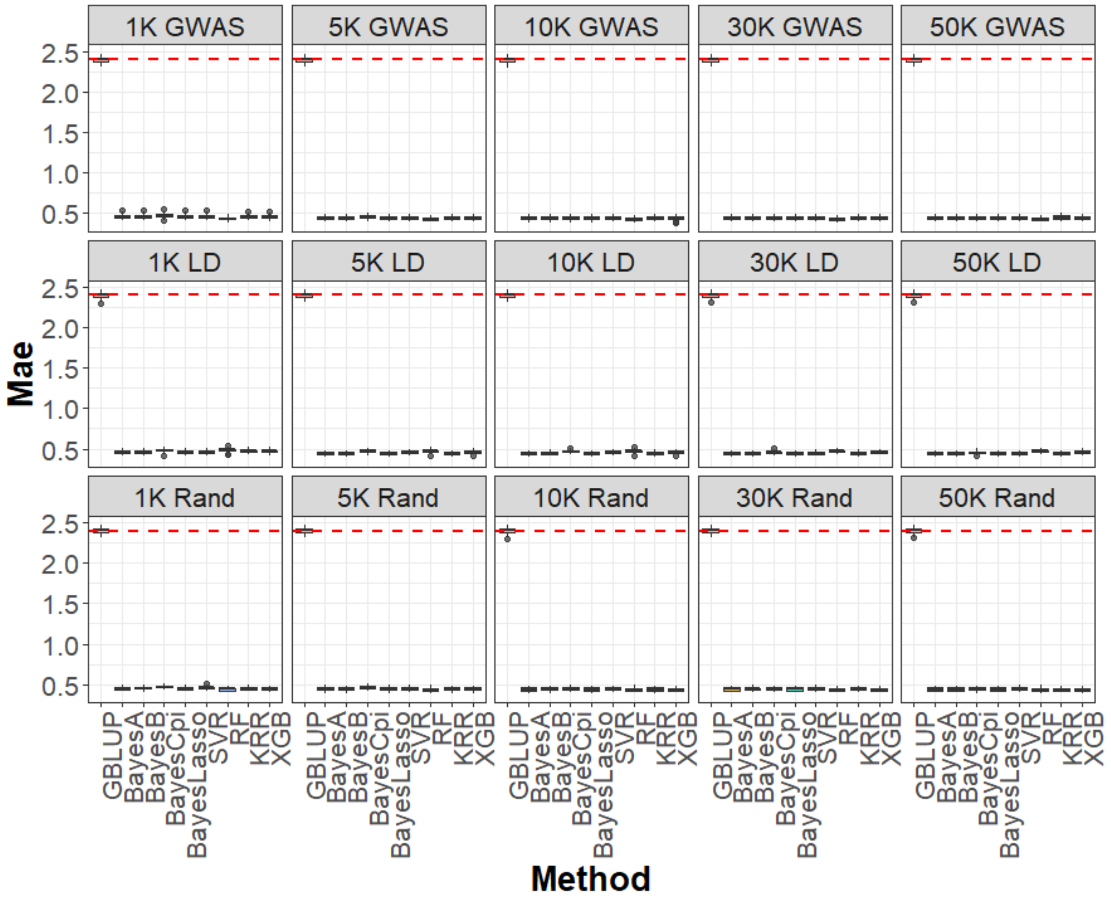

Figure S7 Mean absolute error (Mae) of genomic prediction for caviar color based on GWAS, linkage disequilibrium (LD), and random SNP selection strategies across different SNP densities, using linear and machine learning models.

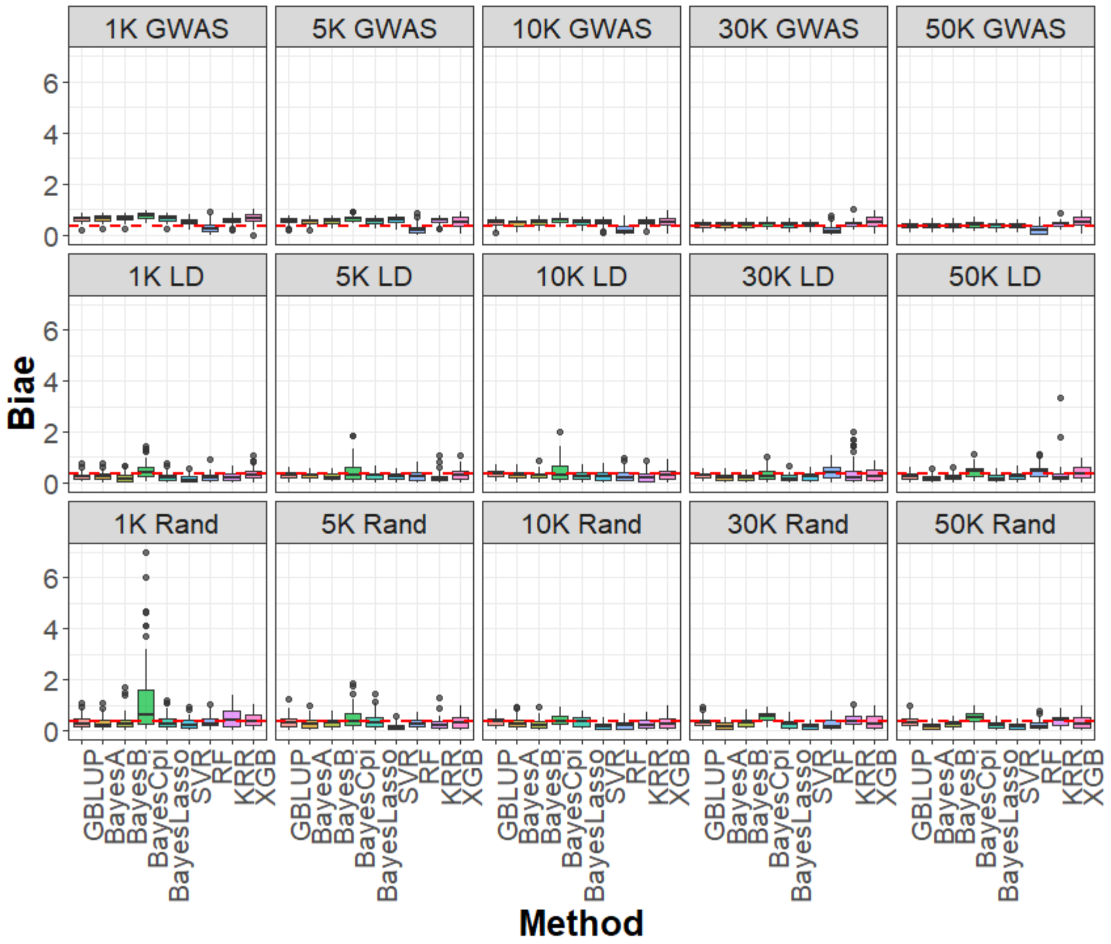

Figure S8 Bias of genomic prediction for body weight based on GWAS, linkage disequilibrium (LD), and random SNP selection strategies across different SNP densities, using linear and machine learning models.

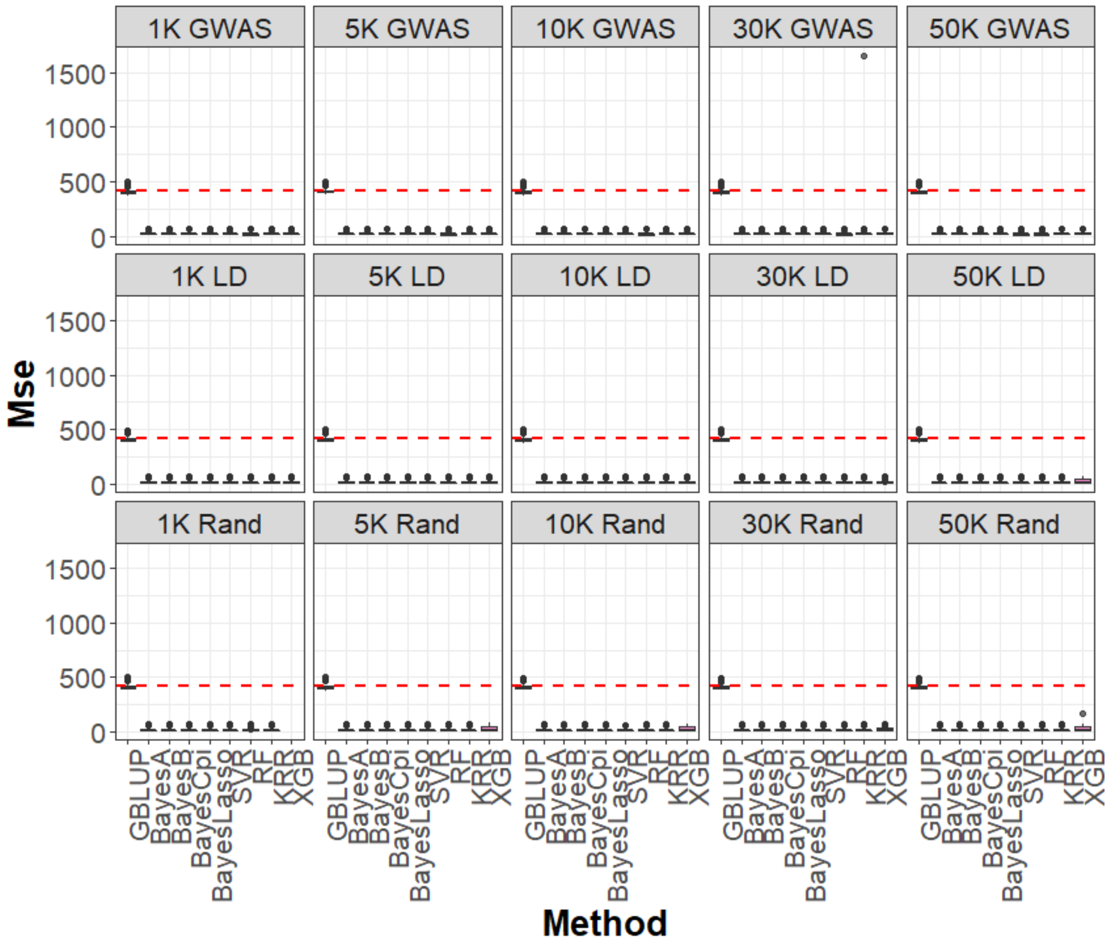

Figure S9 Mean squared error (Mse) of genomic prediction for body weight based on GWAS, linkage disequilibrium (LD), and random SNP selection strategies across different SNP densities, using linear and machine learning models.

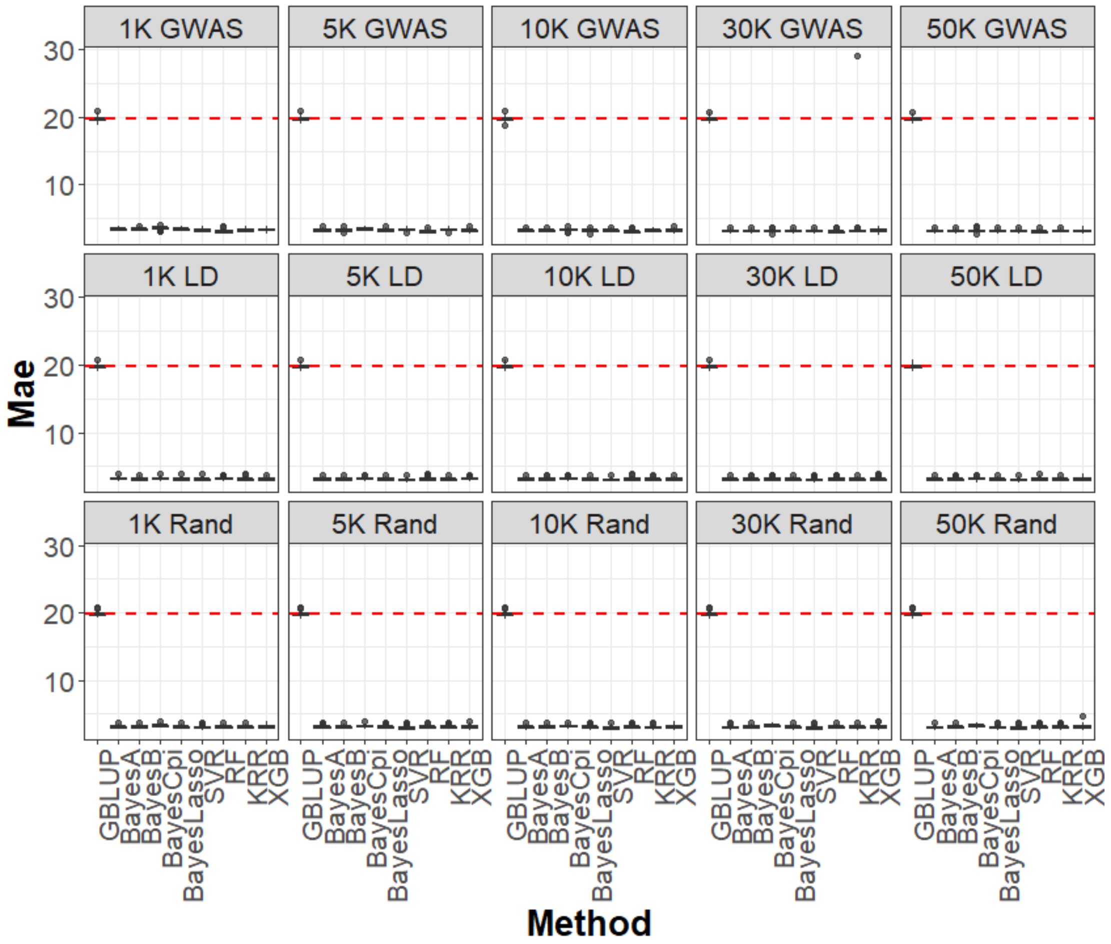

Figure S10 Mean absolute error (Mae) of genomic prediction for body weight based on GWAS, linkage disequilibrium (LD), and random SNP selection strategies across different SNP densities, using linear and machine learning models.
